# Heat-Killed *Lactobacillus paracasei* SMB092 Reduces Halitosis by Stimulating the Expression of β-Defensins in Oral Keratinocytes

**DOI:** 10.3390/microorganisms12112147

**Published:** 2024-10-25

**Authors:** Won-Ju Kim, Gyubin Jung, Taewook Kim, Jinseon Kim, Byung-Serk Hurh, Hangeun Kim, Do Yu Soung

**Affiliations:** 1 Sempio Foods Company, R&D Center, Cheongju 28156, Republic of Korea; kwonju@sempio.com (W.-J.K.); kjinseon@sempio.com (J.K.); hbyungserk@sempio.com (B.-S.H.); 2 Graduate School of Biotechnology, Kyung Hee University, Yongin 17104, Republic of Korea; herbdoctor97@khu.ac.kr; 3 CJ Bioscience Inc., Seoul 04527, Republic of Korea; kay1116@cj.net; 4 Research and Development Center, Skin Biotechnology Center Co., Ltd., Yongin 17104, Republic of Korea; hkim93@khu.ac.kr

**Keywords:** *Lactobacillus paracasei*, oral epithelial cell, mucin-binding protein domain, β-defensin, toll-like receptor, *Porphyromonas gingivalis*, hydrogen sulfide

## Abstract

The purpose of this study is to evaluate *Lactobacillus paracasei* SMB092 as a prophylactic agent for oral pathogens. We examined the physical interaction of SMB092 with a host by identifying the presence of mucus-binding (MuB) protein domains and the capacity of the mucin binding. We determined the role of heat-killed SMB092 in host oral immunity by quantifying the mRNA levels of β-defensins (BDs), Toll-like receptors (TLRs), and their cofactors (CD14/CD36) in normal human oral keratinocytes (HOK-16B cells). To assess the clinically relevant oral health effects of heat-killed SMB092, the growth of *Porphyromonas (P.) gingivalis* and the production of a volatile sulfur compound (H_2_S) were also measured in the filtered condition media (FCM) obtained from its cultures with HOK-16B cells. SMB092 possessed 14 putative MuB protein domains and was attached to mucin. Significant amounts of hBD1/2 and TLR2/6 were expressed in heat-killed SMB092-treated HOK-16B cells. The specific neutralization of TLR2 attenuated the expression of hBD1/2 and CD14/CD36. The FCM inhibited the growth of *P. gingivalis* and the production of H_2_S. Our data indicate that heat-killed SMB092 may contribute to a healthy oral microbiome as an immune stimulant in the production of BDs via the activation of the TLR2/6 signaling pathway.

## 1. Introduction

Periodontal disease is one of the most prevalent oral diseases, posing a significant economic and health burden to people around the world. As people age, one of the main reasons for the incidence of periodontal diseases such as gingivitis and periodontitis is pathogenic bacteria [1]. Halitosis refers to odors originating from the mouth and adjacent organs, causing many people discomfort in their daily lives; pathogenic bacteria are involved in producing the main components of halitosis, volatile sulfur compounds (VSCs). VSCs include hydrogen (H_2_S), methyl mercaptan (CH_3_SH), and dimethyl sulfide (CH_3_SCH_3_). Oral pathogens commonly found in the mouths of patients with periodontal disease and halitosis include *Porphyromonas (P.) gingivalis*, *Fusobacterium (F.) nucleatum*, and *Prevotella intermedia*, which are Gram-negative and anaerobic bacteria [2,3,4].

The long-term use of antibiotics and antiseptics, commonly used as periodontal pathogen treatments, has been associated with side effects such as bacterial resistance and hypersensitivity [5]. As an alternative, there has been significant research into natural substances and probiotics. In particular, probiotics, mainly lactic acid-producing bacteria, have been reported to secrete antimicrobial substances, such as hydrogen peroxide, organic acids, fatty acids, and bacteriocins, that inhibit pathogenic bacteria such as *P. gingivalis* and *F. nucleatum* [6,7]. Furthermore, probiotics with various adhesion proteins, including mucus-binding (MuB) protein domains, are able to bind to the mucus layer for competitive colorization with pathogenic bacteria in establishing the microbiome homeostasis of host oral cavities and guts [8,9]. It has therefore been emphasized that probiotics typically exert beneficial effects on a host by being active and alive, in addition to colonizing oral as well as intestinal tissues [10].

On the other hand, lipoteichoic acid (LTA) on the cell walls of Gram-positive commensal microorganisms has been revealed to play a major role in stimulating epithelial cells to express antimicrobial substances, including β-defensins (BDs) [11]. BDs are small cationic peptides with broad antimicrobial and chemotactic activities, are detected in saliva as well as gingival crevicular fluid, and are essential for maintaining the oral environment [12]. Studies indicate that beneficial microorganisms do not necessarily need to be alive to enhance a host’s defense system, contributing to the host’s health. Nonviable forms of these microbes are easier to handle and can be applied in various fields within the food industry [13]. Therefore, our null hypothesis is that heat-killed *Lactobacillus (L.) paracasei* SMB092 (SMB092) stimulates oral epithelial cells to produce BDs, which prevents the growth of oral pathogens and the development of halitosis.

We have characterized SMB092 isolated from Makgeolli, a traditional Korean fermented rice wine. Its Genbank accession number is CP170332.1 (https://www.ncbi.nlm.nih.gov/nuccore/CP170332, accessed on 30 September 2024). The aim of this study is to find the potential of heat-killed SMB092 as a prophylactic ingredient for oral health maintenance. Thus, to confirm the physical interaction of SMB092 with a host’s oral cavity, we identified whether SMB092 has putative cell adhesion genes targeting mucus-binding (MuB) proteins and is capable of binding to mucus. We also evaluated the effects of heat-killed SMB092 on host oral immunity by measuring the production of antimicrobial peptides (β-defensins: BDs), microbial membrane-bound receptors (Toll-like receptors: TLRs), and their cofactors (CD14/CD36) in primary normal human oral keratinocytes (HOK-16B cells). Additionally, we examined whether the filtered conditioned media (FCM) obtained from HOK-16B cell cultures with heat-killed SMB092 exhibited antimicrobial activity against oral pathogens related to halitosis and periodontal disease.

## 2. Materials and Methods

### 2.1. Bacterial Strains

An SMB092 strain was incubated for 15 h in de Man, Rogosa, and Sharpe (MRS) broth (MB cell, Seoul, Republic of Korea) at 37 °C, and the cultures were spread on MRS agar plates to count the colonies. To evaluate the protective effects of heat-killed SMB092, the aforementioned cultured strain was centrifuged (8000 RPM, 4 °C, 8 min), washed twice with phosphate-buffered saline (PBS; Sigma-Aldrich, St. Louis, MO, USA), and heat-treated in a water bath (85 °C, 30 min). The heat-killed SMB092 strain was suspended in PBS at 9 log cells/mL and diluted to appropriate concentrations with PBS for the study.

*P. gingivalis* was grown in brain heart infusion (BHI) broth media (BD Difco, Bergen County, NJ, USA) at 37 °C under anaerobic conditions. To count the colony, BHI agar plates were used.

### 2.2. Whole-Genome Sequencing Analysis of SMB092

The genome of the SMB092 strain was constructed de novo by using PacBio sequencing data. Sequencing analysis was performed at CJ Bioscience, Inc. (Seoul, Republic of Korea). PacBio sequencing data were assembled with Flye2.9.2. Contigs were rearranged to start with dnaA/repA or a replication origin using Circlator 1.4.0 (Sanger Institute, Hinxton, UK).

The finding of genes and the functional annotation pipeline of whole-genome assemblies used the EzBioCloud (https://www.ezbiocloud.net, accessed on 1 September 2024) genome database. Coding sequences (CDSs) for proteins were predicted by Prodigal 2.6.2 [14]. Genes coding for tRNA were searched for by using tRNAscan-SE 1.3.1 [15]. rRNA and other non-coding RNAs were searched for via a covariance model search with the Rfam 12.0 database [16]. CRISPRs were detected by PilerCR 1.06 [17] and CRT 1.2 [18]. The CDSs were classified into groups based on their roles, with reference to orthologous groups (EggNOG 4.5; http://eggnogdb.embl.de, accessed on 1 September 2024) [19]. For more functional annotation, the predicted CDSs were compared with the Swiss-Prot [20], KEGG [21], and SEED [22] databases by using the UBLAST v8.0.1517 program [23].

### 2.3. Identification of the Putative MuB Responsible for the Adhesion Protein in SMB092

To identify the MuB domain from the predicted CDSs, three domains from the Pfam database—the mucin-binding domain (*MucBP_2*: PF17965), Muc B2-like domain (*Muc_B2*: PF17966), and MucBP domain (*MucBP*: PF06458)—were searched for across all CDSs using the Pfam v37.0 database (Pfam-A.hmm) and HMMER’s hmmsearch (version 3.1b2). An e-value threshold of 1 × 10^−10^ was applied to obtain significant results. If domain locations overlapped within the same gene based on alignment coordinates, the domain with the lower e-value was used. This process was performed using a simple Python (version 3.8.15) script. The results were visualized using DOG 2.0 [24].

### 2.4. Mucin-Binding Assay

Adhesion activity was investigated according to Karbowiak et al. [25], with some modification. Briefly, mucin from bovine submaxillary glands (Sigma-Aldrich, St. Louis, MO, USA) at a concentration of 0.1 mg/mL in PBS was applied at a volume of 1 mL per well of a 24-well plate at 4 °C, overnight. After that, the well was washed twice with PBS. Then, 500 μL of viable or heat-killed SMB092 strain was added to the wells coated with mucin and incubated at 37 °C for 1 h. To remove the unbound bacteria, the wells were rinsed three times with PBS and adhered cells were fixed by heating (60 °C, 20 min). Following treatment with 0.1% crystal violet solution to each well, PBS washing was performed thrice. To quantify the mucin-binding activity of SMB092, each well was treated with 10% acetic acid for 15 min and the absorbance was estimated at 570 nm.

### 2.5. Cell Culture

HOK-16B (human oral keratinocyte) cells were maintained in keratinocyte growth medium (PromoCell GmbH, Heidelberg, Germany) supplemented with gentamicin (final concentration of 30 μg/mL; Thermo Fisher Scientific, Waltham, MA, USA) and amphotericin B (final concentration of 15 ng/mL; Thermo Fisher Scientific, Waltham, MA, USA) at 37 °C under air conditions of a 5% CO_2_ level and 95% humidity [26]. The cells were dissociated with trypsin-EDTA (Welgene, Gyeongsangbuk-do, Republic of Korea) and sub-cultured every 3 or 4 days.

### 2.6. Determination of Cell Viability

The viability of HOK-16B cells was evaluated through a 3-(4,5-dimethylthiazol-2-yl)-2,5-diphenyltetrazolium bromide (MTT) assay [27], with modifications. Briefly, the cells (2.4 × 10^4^ cells/well in 96-well plates) were cultured until monolayer formation. They were treated with heat-killed SMB092 for 24 h in a CO_2_ incubator at 37 °C. Following the addition of the MTT solution at a final concentration of 0.5 mg/mL, the plates were incubated for 30 min. After discarding the supernatant, the resulting formazan deposits of each well were dissolved in dimethyl sulfoxide. The absorbance was estimated at 450 nm, and it was judged that there was no significant cytotoxicity when the survival rate was more than 90% compared to the negative control group (none).

### 2.7. Measurement of mRNA Expression

The expression levels of the mRNA of antimicrobial peptides and receptors were examined via a real-time polymerase chain reaction [28]. HOK-16B cells were seeded at 2.4 × 10^4^ cells/well in a 6-well plate. After overnight incubation, the wells were treated with 1.0 × 10^4^, 1.0 × 10^5^, 1.0 × 10^6^, 1.0 × 10^7^, and 1.0 × 10^8^ cells/mL of SMB092 for 1 h. Following washing with PBS and incubation for another 1 h with antibiotic-free media, the cell supernatant was removed. To determine the signaling mechanism, polyclonal antibody to human TLR2 (PAb-hTLR2; InvivoGen, San Diego, CA, USA), a neutralizing antibody, was added to the cells at a concentration of 5 μg/mL for 30 min before 1 h of treatment of heat-killed SMB092. The cells were then washed once with PBS. After adding new media, they were incubated for another hour. Total RNA was extracted using an easy-BLUE™ Total RNA Extraction Kit (iNtRON Biotechnology, Gyeonggi-do, Republic of Korea). The complementary DNA (cDNA) was synthesized using PrimeScript™ RT Master Mix (TaKaRa Bio Inc., Shiga, Japan), according to the manufacturer’s instructions. TB Green^®^ Premix Ex Taq™ II (TaKaRa Bio Inc., Shiga, Japan) and an AriaMx Real-time PCR System (Agilent, Santa Clara, CA, USA) were used to perform real-time PCR. The expression of glyceraldehyde-3-phosphate dehydrogenase (GAPDH) mRNA served as an internal reference. The primer sets used in this study are listed in Table 1.

### 2.8. Bactericidal Assay

The antibacterial activity of the FCM obtained from HOK-16B cells treated with heat-killed SMB092 was examined as previously described, with modifications [29]. Briefly, HOK-16B cells (2.4 × 10^4^ cells/well in a 6-well plate) were cultured overnight to stabilize them. Then, the cells were treated with SMB092 at concentrations of 1.0 × 10^6^ or 1.0 × 10^8^ cells/mL. The cells were incubated at 37 °C for 1 h, followed by treatment with 2 mL of an antibiotic-free medium to each well for another 2 h. The cell culture supernatants were collected by centrifugation (3000 rpm, 4 °C, 10 min) and filtered through a 0.45 μm pore size filter to prepare the FCM. The FCM was concentrated to one-fourth of the original volume by using a SpeedVac (Eppendorf, Hamburg, Germany). To determine the antibacterial activity of the FCM, 500 μL of the concentrated FCM was mixed with 1.0 × 10^4^ CFU/mL *P. gingivalis* and incubated at 37 °C for 6 h. Subsequently, 100 μL of the reaction mixture was spread on BHI agar plates and incubated anaerobically at 37 °C for 2 days. The number of colonies formed on the BHI agar plates was counted. Additionally, 50 μg/mL of gentamycin (GEN) was used as a positive control.

### 2.9. Measurement of H_2_S

To determine whether the FCM exhibits beneficial potential to reduce halitosis, the production of H_2_S was quantified [30]. The test media (growth medium containing 0.1% cysteine, 0.2% FeSO_4_, and 0.1 M 2-morpholinoethanesulphonic acid; pH 7.0) was added to the mixture of FCM and *P. gingivalis* mentioned in Section 2.8. Following incubation at 37 °C for 2 days, absorbance was measured at 700 nm.

### 2.10. Statistical Analysis

The experimental results are presented as the mean ± standard deviation (SD). The statistical analysis of the experimental data was performed using a *t*-test or one-way ANOVA (Statistical Package for the Social Science (SPSS), Ver. 12.0, SPSS Inc., Chicago, IL, USA), and the significance was tested at levels of *p* < 0.05, *p* < 0.01, and *p* < 0.001.

## 3. Results

### 3.1. General Genome Features of SMB092

**Figure 1:**
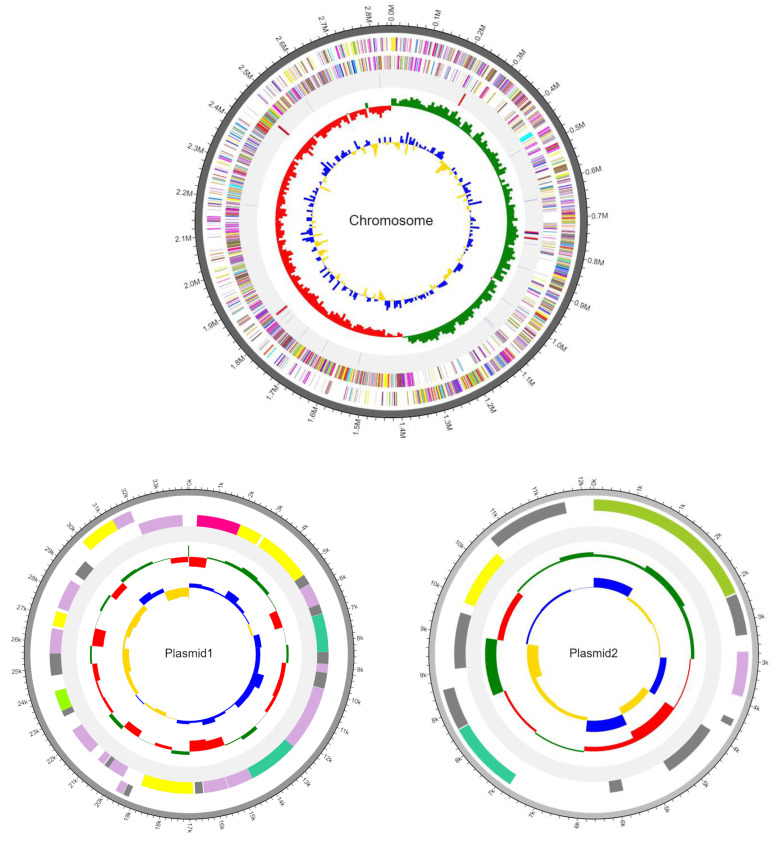
Circular gene map of SMB092. Each circle from the outside to the center indicates CDSs on the forward strand, CDSs on the reverse strand, tRNA, rRNA, GC skew, and GC content. The genomic mean GC skew value is used as the baseline, relative to which higher-than-average values are displayed in green, whereas lower-than-average values are displayed in red. The GC ratio also uses the genomic mean GC ratio value as its baseline, with higher-than-average values in blue and lower-than-average values in yellow.

The genome assembly of SMB092 consists of a 2.849 Mbp circular chromosome, a 34.051 Kbp plasmid1, and a 11.636 Kbp plasmid2, as shown in Figure 1 and Table 2. The size of the entire genome sequence of SMB092 was 2,895,229 bp. The average G + C content of the whole genome was 46.3%. The GC content of each contig was 46.3% (chromosome), 39.2% (plasmid1), and 40.5% (plasmid2). Additionally, 2802 CDSs were identified in the total genome of SMB092, specifically 2754 CDSs for chromosome, 36 CDSs for plasmid1, and 12 CDSs for plasmid2. The SMB092 strain contained 59 tRNA and 15 rRNA genes.

### 3.2. Defining Genes Encoding MuB and Mucin-Binding Activity of SMB092

**Figure 2:**
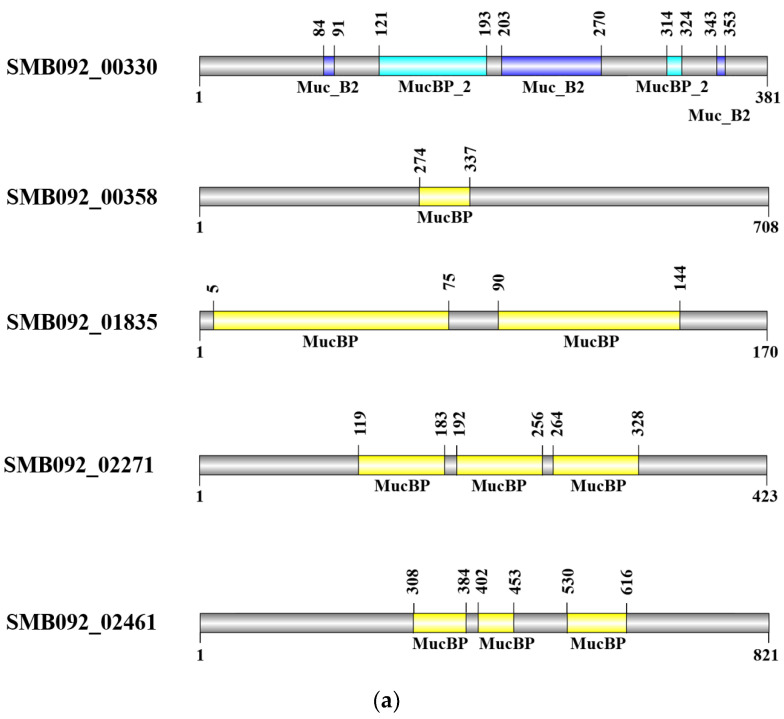

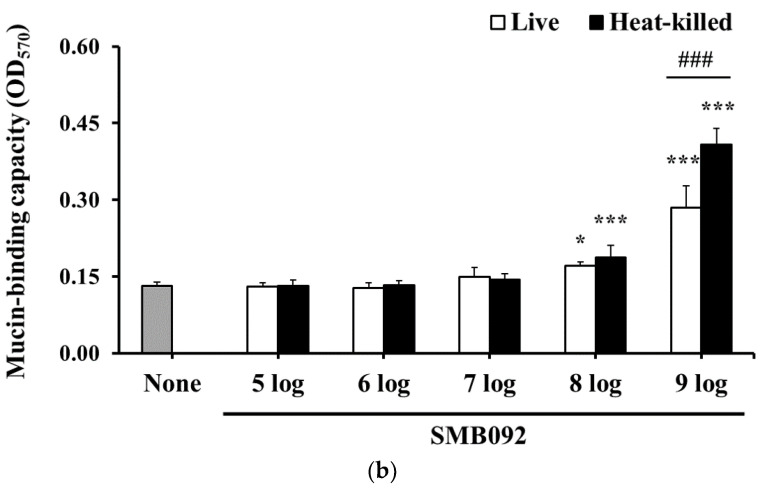
Architecture of the genes encoding MuB protein domains and the mucin-binding activity of SMB092. (**a**) MuB protein domain containing sequences in SMB092. (**b**) Mucin-binding activity of live and heat-killed SMB092. * *p* < 0.05; *** *p* < 0.001 compared to none. ^###^ *p* < 0.001 compared to alive.

We searched for genes encoding for MuB proteins that allow SMB092 to bind to mucin (Figure 2a). Mucin-binding domain (*MucBP_2*), Muc B2-like domain (*Muc_B2*), and MucBP domain (*MucBP*) were searched for across all CDSs, and a total of 14 domains were identified in five CDSs from the chromosome—SMB092_00330, SMB092_00358, SMB092_01835, SMB092_02271, and SMB092_02461. Three *Muc_B2* domains and two *MucBP_2* domains were identified in SMB092_00330. SMB092_00358, SMB092_01835, SMB092_02271, and SMB092_02461 contained one, two, three, and three MucBPs, respectively. No MuB proteins were observed in CDSs from the plasmids. This indicates that SMB092 is likely to interact directly with a host via the mucin-binding protein domain.

The results of in vitro adhesion to mucin studies are presented in Figure 2b. Viable and heat-killed SMB092 exhibited dose-dependent adhesion ability to mucin from bovine submaxillary glands. In particular, 10^9^ cells/mL of heat-killed SMB092 showed higher binding activity than 10^9^ CFU/mL of viable SMB092. These results indicate that SMB092 interacts with host cell mucins despite being inactivated by heat treatment.

### 3.3. The Effect of SMB092 on Cytotoxicity and BD Production in HOK-16B Cells

**Figure 3:**
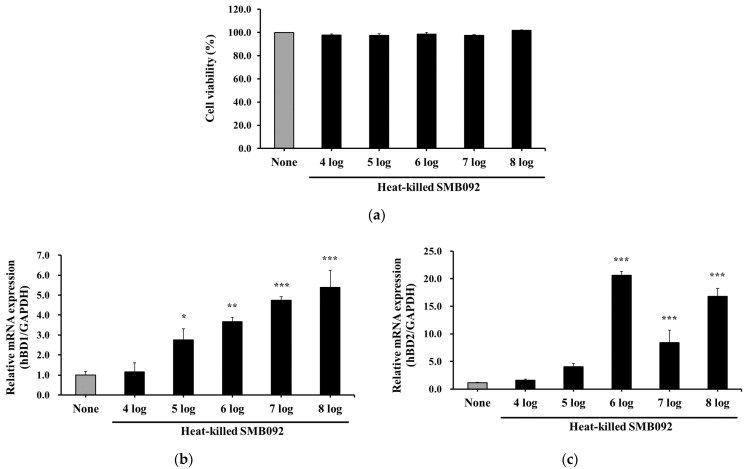
The effect of SMB092 on cytotoxicity and hBD production in HOK-16B cells. (**a**) Viability of HOK-16B cells via heat-killed SMB092; (**b**) hBD1 production in heat-killed SMB092-treated HOK-16B cells; and (**c**) hBD2 production in heat-killed SMB092-treated HOK-16B cells. * *p* < 0.05, ** *p* < 0.01, and *** *p* < 0.001 compared to none.

To investigate the safety and ability to express hBDs of heat-killed SMB092 for oral health, we utilized human primary normal oral keratinocytes (HOK-16B cells). The tested strain at concentrations below eight log cells/mL did not show any cytotoxicity in HOK-16B cells (Figure 3a); therefore, in subsequent experiments, the influence of cytotoxicity was not contemplated.

Compared to the untreated negative control group (none), the production of hBD1 was increased markedly by treatments with 10^5^, 10^6^, 10^7^, and 10^8^ cells/mL SMB092 (Figure 3b). In particular, the expression levels of hBD1 were increased dose-dependently. Furthermore, 10^6^, 10^7^, and 10^8^ cells/mL heat-killed SMB092 also significantly induced mRNA levels of hBD2 (Figure 3c). This indicates that heat-killed SMB092 has the ability to stimulate a host for antimicrobial peptide production.

### 3.4. The Effect of SMB092 on the Expression of TLRs in HOK-16B Cells

**Figure 4:**
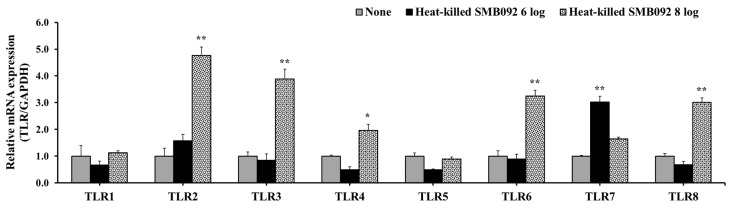
The effect of SMB092 on the expression of TLRs in HOK-16B cells. * *p* < 0.05; ** *p* < 0.01 compared to none.

To examine the regulation of LTA-sensing receptors by heat-killed SMB092, we measured TLRs in HOK-16B cells. As shown in Figure 4, 10^8^ cells/mL of heat-killed SMB092 significantly upregulated the expression of TLR2, TLR3, TLR4, TLR6, and TLR8. The data suggest that heat-killed SMB092 induces the expression of TLRs and LTA-sensing receptors.

### 3.5. The Effect of TLR2 Neutralization on the Expression of hBD1/hBD2 and CD14/CD36 in HOK-16B Cells

**Figure 5:**
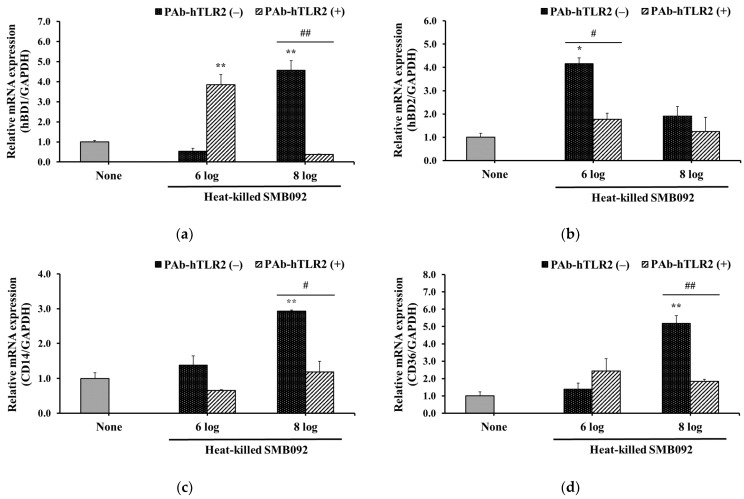
The effect of TLR2 neutralization on the expression of hBDs and CD14/CD36 in HOK-16B cells. mRNA expression levels of (**a**) hBD1 and (**b**) hBD2; mRNA expression levels of (**c**) CD14 and (**d**) CD36. * *p* < 0.05; ** *p* < 0.01 compared to none. None: Negative control. ^#^ *p* < 0.05; ^##^ *p* < 0.01 compared to PAb-hTLR2 (−).

To verify the signaling pathway, the relative expression levels of hBD1/2 mRNA induced by heat-killed SMB092 were measured in HOK-16B cells treated with a TLR2-neutralizing antibody (Figure 5a,b). Compared to the condition without TLR2 neutralization, 10^8^ cells/mL of heat-killed SMB092 decreased hBD1 expression. In the case of hBD2, mRNA expression levels were significantly reduced following treatment with the polyclonal antibody (PAb-hTLR2) in the six log heat-killed SMB092 group.

Furthermore, we determined the effect of TLR neutralization on the expression of CD14/CD36. As shown in Figure 5c,d, 10^8^ cells/mL of heat-killed SMB092 significantly induced the expression of CD14 and CD36. However, TLR2 neutralization markedly reduced the expression levels of CD14 and CD36 induced by SMB092.

The results showed that TLR2, the membrane receptor of the host epithelial cells, is a key factor in the production of BDs and their cofactor, CD14/CD36.

### 3.6. Effects of the FCM on P. gingivalis and Its Mediated H_2_S Production

**Figure 6:**
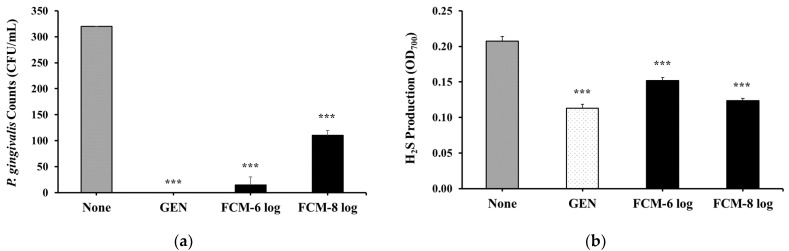
The inhibitory survival of *P. gingivalis* and the reduced production of H_2_S by the FCM. (**a**) Viability of *P. gingivalis*; (**b**) H_2_S production. *** *p* < 0.001 compared to none. None: Negative control.

The results for the evaluation of the antimicrobial effect of the FCM containing BDs secreted by HOK-16B cells are shown in Figure 6a. As expected, 50 μg/mL gentamycin (GEN) completely inhibited the growth of *P. gingivalis*. Additionally, the FCM obtained from the culture of HOK-16B cells treated with both 10^6^ cells/mL and 10^8^ cells/mL SMB092 significantly reduced the colony counts of *P. gingivalis*.

Figure 6b shows the production of H_2_S by *P. gingivalis*. The FCM in GEN significantly exhibited 52% inhibitory effects on H_2_S, a VSC, produced by *P. gingivalis*. The FCM obtained from the culture of HOK-16B cells treated with both 10^6^ and 10^8^ cells/mL of SMB092 showed 29% and 43%, respectively, compared to none. These results suggest that the antimicrobial substances, including BDs, secreted by oral epithelial cells stimulated with heat-killed SMB092 reduced the growth of *P. gingivalis* and its mediated H_2_S concentration.

## 4. Discussion

The oral cavity harbors extensive microbiota including commensal and pathogenic bacteria. It is constantly covered with saliva containing antimicrobial peptides produced by oral epithelial tissues. Saliva plays a major role in maintaining the homeostasis between the oral microbiota and the host [31]. It is an important strategy to strength the host’s oral mucosal barrier by stimulating the expression of the defense factors, such as BDs, in the saliva. In this study, we first characterized and identified the *MuB* gene encoding the mucin-binding protein domain in SMB092 and measured its mucin-binding ability. We then elucidated the ability of SMB092 to induce the expression of antimicrobial peptides in gingival epithelial cells and consequently improve oral health.

The mucin-binding domains on commensal bacteria are crucial for interactions with host extracellular matrix proteins and epithelial cells to interfere with the colonization of pathogens and initiate intracellular signaling pathways [32,33]. Identifying the structural properties of mucin-binding domains and the capacities with mucin would be key factors with which to determine the mechanisms behind the probiotic–host interaction. In this study, we confirmed that SMB092 possesses 14 putative *MuB* genes encoding the mucus-binding domains (Figure 2a). We also found that, regardless of the SMB092 live status, it is able to bind the mucus layer. Previous studies have identified that *Lactobacillus* strains differ in the size and number of mucus-binding domains [9,34,35]. Nishiyma et al. [35] demonstrated that the mutation of the mucus-binding factor (*mbf*) in the *Lactobacillus rhamnosus* FSMM22 strain significantly reduced their adhesive capacity to porcine colonic mucin and other extracellular matrix proteins, such as laminin, fibronectin, and collagen IV. *Lactobacillus reuteri* cell- and mucus-binding protein A (CmbA) also attaches to intestinal epithelial cells as well as mucus [36]. Our findings suggest that the dynamics of the mucus-binding domain on SMB092 impact its interaction with the host oral cavity for regulating pathogens, initiating downstream signaling pathways, and modulating host immune responses.

The commensal bacteria are known to play an important role in strengthening a host’s mucosal barrier by stimulating epithelial tissues to express antimicrobial factors [37]. We showed that the expression levels of hBD1 and hBD2 were upregulated following treatment with heat-killed SMB092 in HOK-16B cells (Figure 3b,c). Similar to our data, heat-killed *Lactobacillus casei* and *L. fermentum* were stimulated to express mRNA levels of hBD2 comparably with their respective live probiotics in Caco-2 human epithelial cells [11]. *Lactobacillus helveticus* SBT2171, a live form, also upregulated mRNA levels of BD2 and BD3 in human gingival epithelial Ca9-22 cells [38]. Additionally, various probiotic *Lactobacillus* strains stimulated human gingival epithelial cells to induce the secretion of either BD2 or BD4 [39]. Our and other results indicate that the commensal bacteria, regardless of their live status, as the approach to probiotics have sufficient conditions with which to stimulate epithelial cells to produce BDs.

The molecular signaling pathway behind BD production by commensal bacteria has not yet been clearly understood; however, we demonstrated that heat-killed SMB092 stimulated the expression of host transmembrane receptors, particularly TLR2 and TLR6, as it is well known for recognizing LTA (Figure 4). Furthermore, we verified that the induction of hBD1/2 and TLR accessary molecules, CD14/CD36, by heat-killed SMB092 was inhibited by the presence of TLR2-specific antibodies (Figure 5). Kim et al. [40] reported a similar finding, that LTA of *Lactobacillus* strains is one of main factors in BD production. The expression of BDs by commensal bacteria has also been associated with the nuclear factor-kappa B (NF-κB) signaling pathway through the interaction of their LTA with pattern recognition receptors, such as TLR2/6 in epithelial cells [41]. Additionally, the mRNA levels of CD14 were downregulated in the absence of TLR2 using genetically modified mice [42]. Thus, the molecular mechanism in the expression of hBD1 and hBD2 by heat-killed SMB092 is likely an intracellular downstream pathway via the interaction of its LTA with TLR2/6 on the membrane of human oral keratinocytes.

The principle of hBDs as antimicrobial agents has been described as the binding of positively charged hBDs to negatively charged components of pathogenic microbial cell membranes, disrupting and leaking their integrity [43]. We demonstrated that the growth of *P. gingivalis* was inhibited when it reacted with the FCM obtained from the incubation of heat-killed SMB092 with human oral keratinocytes (Figure 6a). Sequentially, the FCM significantly reduced *P. gingivalis*-mediated H_2_S production (Figure 6b). The antimicrobial effects of the FCM are probably due to the hBD1 and hBD2 expressed by human oral keratinocytes cultured with heat-killed SMB092. The possible role of BDs in oral health has been reported as the concentration of hBD2 in saliva being inversely related to the occurrence of gingivitis and the production of H_2_S in human subjects [44,45]. In vitro studies have also shown that hBD2 inhibited detrimental oral bacteria, such as *F. nucleatum* and *P. gingivalis*, in a dose-dependent manner [46]. *Lactobacillus paracasei* LMT18-32 itself produced antimicrobial substances, such as bacteriocins, that inhibited the growth of *P. gingivalis* [47]. Another *L. paracasei* ET-22 strain exhibited a significant inhibitory effect on biofilms of the oral pathogen *S. mutans* in all of its live, heat-killed, and secreted forms [48]. Furthermore, in patients with halitosis, the oral intake of live and heat-killed *L. paracasei* ET-22 for 28 days lead to a reduction in VSC levels in the oral cavity [49]. Although it has been reported that the mitigation of halitosis by heat-killed ET-22 is attributed to the marked inhibition of pathogens, including Rothia and Streptococcus, at the genus level, there are no reports of an association between halitosis and Rothia as well as Streptococcus [50,51]. On the other hand, we did not focus on the ability of SMB092 itself to produce antimicrobial substances. Instead, we demonstrated that SMB092, even when not alive, stimulated oral keratinocytes to produce BDs, which could be an important factor in preventing *P. gingivalis*-mediated periodontal diseases and halitosis, thereby maintaining oral health.

Future research should focus on (1) the characterization of other adhesion genes in SMB092, (2) the determination of its binding ability to human oral keratinocytes, (3) the confirmation of BD protein concentrations in the FCM, and (4) the investigation of other antimicrobial factors in the FCM. Finally, preclinical studies and clinical trials need to be conducted to validate these findings on the full therapeutic potential of SMB092 in various oral health applications.

## 5. Conclusions

Based on the data of whole-genome sequences, the putative mucus-binding domains were identified in SMB092. SMB092 remarkably adhered to mucin, regardless of its live status, and had no significant cytotoxicity to human oral keratinocytes. The heat-killed SMB092 upregulated the mRNA levels of antimicrobial peptides (hBD1/2), as well as their regulators (TLR2/6) and cofactors (CD14/CD36) in the same cell line. The FCM obtained from the incubation of heat-killed SMB092 with HOK-16B cells inhibited the growth of *P. gingivalis* and its mediated production of H_2_S. Overall, the heat-killed SMB092 could be employed as a prophylactic agent for the improvement of oral hygiene, including the treatment of halitosis and periodontal diseases.

## Figures and Tables

**Table 1:** Primer sets used in this study.

Primers	Forward	Reverse
hBD1	CTGCCTGCCCGATCTTTACC	CACTCCCAGCTCACTTGCAG
hBD2	CATCAGCCATGAGGGTCTTGT	ACAGGATCGCCTATACCACCA
TLR1	CTTCTGTTTTTGTGGCCAGGG	GGTGCCCAATATGCCTTTGTTA
TLR2	TTCCCTGGGCAGTCTTGAAC	GGCTTGAACCAGGAAGACGA
TLR3	CGGCCAACACTGTGAATGTG	CAGTGTCTCAGCTGTCAGGG
TLR4	TGGTGTCCCAGCACTTCATC	TGATACCAGCACGACTGCTC
TLR5	ACTGACAACGTGGCTTCTCC	GGGCAACTATAAGGTCAGGGG
TLR6	TGCAGACAAGTGTGAGGGAC	CTGAACCTGTGTGCTCCTGT
TLR7	TTCCCACGAACACCACGAAC	AGTCTGTGAAAGGACGCTGG
TLR8	TGCCACTGTGACTAATGGTCC	AAAACCGAACGCAACCACAG
CD14	CCTCAAGGAACTGACGCTCG	AGTGCAAGTCCTGTGGCTTC
CD36	GGACGCTGAGGACAACACA	AAGTTGTCAGCCTCTGTTCCA
hGAPDH	AAGAAGGTGGTGAAGCAGGC	TGGGTGTCGCTGTTGAAGTC

**Table 2:** Results of annotation.

Contig Name	Length (bp)	GC Content (%)	CDS	tRNA	rRNA
Chromosome	2,849,542	46.3	2754	59	15
Plasmid1	34,051	39.2	36	0	0
Plasmid2	11,636	40.5	12	0	0
Total	2,895,229	46.3	2802	59	15

## Data Availability

The original contributions presented in the study are included in the article, further inquiries can be directed to the corresponding author.
